# Barriers and facilitators for the utilisation of psycho-oncological services in German hospitals as perceived by patients and healthcare professionals: a mixed-methods study

**DOI:** 10.1186/s12913-025-13053-5

**Published:** 2025-07-01

**Authors:** Liv Betker, Kristina Buch, Pia Berlin, Anna J. Pedrosa Carrasco, Jorge Riera Knorrenschild, Carola Seifart, Pia von Blanckenburg

**Affiliations:** 1 Research Group Clinical Psychology and Psychotherapy, Philipps-University Marburg, Marburg, Germany; 2 Department of Primary Care, Philipps-University Marburg, Marburg, Germany; 3 Research Group Medical Ethics, Philipps-University Marburg, Marburg, Germany; 4 Department of Haematology, Oncology and Immunology, University Hospital of Giessen and Marburg, Marburg, Germany

**Keywords:** Barriers, Cancer patients, Clinical pathway, Facilitators, Mixed-methods, Psycho-oncology

## Abstract

**Background:**

Psycho-oncological services (POS) are an integral and widely recommended part of comprehensive cancer care. However, their utilisation appears to fall short of the perceived need. This study aimed to explore barriers and facilitators for the uptake of POS in the context of existing clinical structures and pathways to POS.

**Methods:**

A mixed-methods study was conducted, drawing on elements of a Delphi approach, including an iterative two-round feedback process and an expert panel. The expert panel consisted of healthcare professionals in cancer care (*n* = 27) and cancer patients (*n* = 14). The first round comprised open-ended questions to explore different perspectives related to the research question. The resulting material was analysed qualitatively and grouped into themes, which were rated according to their perceived importance by the expert panel in the second survey-round (*N* = 27).

**Results:**

The expert panel identified 69 aspects influencing the uptake of POS; 81% were rated as relevant in the second round. They were grouped into structural factors at hospital level, aspects related to internal processes, and factors at patient level. Central aspects were recommendations of POS by the clinical staff, personal introduction of the psycho-oncologist, integration and acceptance of POS within the hospital organisation, information dissemination about POS to both patients and clinical staff, and the possibility of flexible access routes to POS since patients’ preferences differed. Patient-groups more difficult to reach with a POS-offer were also identified.

**Conclusion:**

The results can be used to review implemented clinical pathways to POS in diverse hospital contexts, helping to identify and improve critical aspects accordingly, and thus improve service accessibility.

**Trial registration:**

This study was pre-registered at the German Clinical Trials Register (DRKS-ID: DRKS00025105; registration date: 26-05-2021).

**Supplementary Information:**

The online version contains supplementary material available at 10.1186/s12913-025-13053-5.

## Introduction

Cancer patients often experience significant psychological distress [1, 2]. The diagnosis and uncertainty surrounding prognosis, treatment-related challenges, or financial and practical concerns can contribute to the psychological burden [3]. The field of psycho-oncology has evolved to address these psychological and social aspects of cancer care, improving patients’ well-being and quality of life [4]. Thus, psycho-oncological services (POS) have become integral to comprehensive cancer care. In Germany, the importance of psycho-oncological support is recognised and mandated in national clinical guidelines [5], and the provision of POS is imperative for certification as a cancer centre [6].

However, there is a discrepancy between recommendations, implementation, and utilisation of these services: Although clinically significant psychological distress of cancer patients is reported to be as high as 22–52% [1, 2], the overall uptake of POS lies around 9% [7, 8].

One explanation is that many cancer patients decline the support-offer [9], which previously led to investigations of patient characteristics influencing the uptake of POS [10, 11]. While it is important to consider individual factors, examining contextual and structural conditions that influence the utilisation of POS is equally crucial, especially considering the evidence indicating a gap between desired and received psychological support by cancer patients [12, 13]. The uptake of POS was lowest in acute care hospitals [13]. This highlights the need to investigate contextual factors that may contribute to this discrepancy, especially in the hospital setting.

In Germany, the clinical pathway outlined in the national clinical guidelines since 2014 [5, 14] aims to ensure that patients in need of POS receive the appropriate support offer. It comprises different elements such as continuous distress screening for all cancer patients, provision of information about POS, psycho-oncological diagnostics and referral to appropriate support services and interventions, as well as the evaluation of the interventions provided. Despite these efforts, challenges in the uptake of POS seem to persist [13]. Therefore, exploring barriers and facilitators within the implemented clinical pathways to POS is essential and the aim of the present study.

## Methods

### Study design and procedure

A mixed-method study was conducted, drawing on elements of a modified Delphi approach by incorporating elements such as an iterative feedback process over two rounds with a progression from open-ended questions to a quantitative assessment based on group results and an expert panel [15].

Thus, the strengths of a modified Delphi approach were utilised, including a structured yet open exploration of the topic and the integration of diverse stakeholders’ perspectives, but without the goal of reaching a consensus. The aim was rather to gain insights into the currently employed clinical pathway and to use the feedback system to weigh the reported facilitators and barriers according to their importance for the uptake of psycho-oncological services.

The present study consisted of two rounds: Round1 began with the assessment of demographics, and participants’ experience in the research field, before transitioning to its core element: open-ended questions designed to capture as many aspects as possible regarding barriers and facilitators to psycho-oncological services. The answers were analysed qualitatively. The identified themes of barriers and facilitators were presented in Round2 and rated according to their perceived importance.

The according surveys were developed for the purpose of this study. The surveys for Round1 were developed by the research team, drawing on their expertise in (psycho-)oncological care (perspectives of psychologists and physicians) and informed by existing studies with similar research questions or methodological approaches [16, 17]. The surveys for Round2 were derived from the results of Round1. Pretests were conducted to evaluate the comprehensibility and user-friendliness of the surveys (*n* = 2 laypeople and *n* = 2 independent researchers), the feedback received was used to refine the surveys. The final surveys are provided in full length in the Supplementary Material A.

Both rounds were conducted as online surveys using the platform Unipark [18]. They were carried out between April and July 2021. MAXQDA [19] was used for the qualitative and SPSS [20] for the quantitative analyses.

### Composition of the expert panel

We included two groups of experts to comprehensively answer our research questions: (1) professional experts and (2) experts by lived experience [21]. In our research setting this meant (1) healthcare professionals with working experience in cancer care in hospitals, thereby having direct or indirect contact with POS; (2) cancer patients, due to their lived experiences with cancer and thus clinical pathways in cancer care, including POS. Participants had to be at least 18 years old, be fluent in German, and have their working place or cancer treatment in Germany. Healthcare professionals were recruited via mailing lists from cancer care associations; cancer patients through self-help groups and social media. A snowball sampling approach was employed for both expert groups.

### Surveys

#### Round 1

Two slightly different surveys were formulated, due to different insights of healthcare professionals and cancer patients into the clinical pathway to POS. The surveys had the same overarching questions and shared the same basic structure: (1) demographic and further personal data (either concerning their working field or information about the cancer diagnosis); and (2) two to three open-ended questions concerning barriers and facilitators for the uptake of POS. Here, healthcare professionals were asked to describe (a) aspects that hinder the utilisation of POS; (b) aspects contributing to a higher utilisation of POS; and (c) if they perceive any structurally disadvantaged patient groups in the uptake of POS. The questions for cancer patients were tailored to three subgroups based on their utilisation of POS (users, deliberate non-users, and non-users wishing to have used POS). It covered two aspects: (a) what facilitated their uptake of POS; (b) what conditions made it difficult to access POS, or in case they could not use it, what they would have wished for to utilise it.

#### Round 2

In Round2, the results of the qualitative analysis of Round1 were presented. The research team summarised the findings and prepared them as statements, each describing aspects influencing the uptake of POS. Participants could then rate those aspects according to their perceived relevance. The items were grouped according to their overarching themes. All items in a thematic group started with the same beginning, e.g. “For the uptake of psycho-oncological services (in the hospital), I perceive as hindering” “… a lack of information dissemination about psycho-oncological services (e.g., the service is unknown, patients are not made aware of it)”. The given examples were paraphrased coded segments provided by participants in Round1. The statements were rated on a Likert scale ranging from 1 (totally disagree) to 9 (totally agree).

The surveys for healthcare professionals (33 statements) and patients (16 statements) differed due to their distinct perspectives on the barriers and facilitators to POS reported in Round 1. Accordingly, the qualitative analysis of the data of Round1 led to the identification and synthesis of different themes for patients and healthcare professionals, which in turn resulted in distinct statements for each group in Round2.

### Data analysis

#### Round 1

The material from open-ended questions was analysed using content analysis [22]. Two researchers (LB and KB) coded the material independently. A coding system with inductively derived themes was developed and iteratively expanded. The different themes and subthemes were then grouped into overarching themes based on the research question. The material was recoded using the final coding scheme. Interrater-agreement was assessed using Cohen’s Cappa [23] and differences in coding were discussed until a consensus was reached. Data from healthcare professionals and cancer patients were analysed separately.

#### Round 2

Data from Round2 were analysed quantitatively using descriptive statistics. As interpretation aid, mean rating scores of > 5 were categorised as agreement with the statement (indicating a perceived relevance of the according barrier or facilitator), and ratings < 5 as disagreement; a value of 5 meant a neutral position to the statement (based on the verbal anchors of the Likert-scale).

## Results

### Participants

In Round1, the expert panel comprised 41 participants (*n* = 27 healthcare professionals and *n* = 14 cancer patients). 65.9% (*N* = 27; *n* = 13 healthcare professionals and *n* = 14 cancer patients) of the participants completed Round2. Healthcare professionals (*M*_age_=46.63 [*SD* = 12.49; range:26–69]; 77.8% female) mainly worked as psycho-oncologists (81.5%), the remaining as physicians. The patients (*M*_age_=58 [*SD* = 11.83; range:29–71]; 50.0% female) had different cancer types, and the majority had utilised POS during their illness trajectory (78.6%). Participants lived in eight (healthcare professionals) to ten (patients) different German states, thus representing a range of different hospital experiences. For a full description of the participants’ characteristics, see Table 1.

**Table 1:** Characteristics of the expert panel

Healthcare professionals	Round1 (*N* = 27) *n* (%)	Round2 (*N* = 13) *n (%)*
Mean age in years (*SD*; range)	46.63 (12.49; 26 - 69)	48.46 (11.5; 27–62)
Gender		
Female	21 (77.8)	12 (92.3)
Male	6 (22.2)	1 (7.7)
Educational level		
University degree	27 (100)	13 (100)
Professional background		
Physician	5 (18.5)	2 (15.4)
Psycho-oncologist	22 (81.5)	11 (84.6)
Mean years of working experience (SD; range)	12.26 (10.19, 0 - 35)	10.69 (7.49; 1 - 23)
Part of the psycho-oncological team		
Yes	21 (77.8)	9 (69.2)
No	6 (22.2)	4 (30.8)
Cancer patients	Round1 (*N* = 14) *n* (%)	Round2 (*N* = 14) *n (%)*
Mean age in years (*SD*^a^; range)	58 (11.83; 29–71)	58 (11.83; 29–71)
Gender		
Female	7 (50)	7 (50)
Male	7 (50)	7 (50)
Educational level		
Secondary school	9 (64.3)	9 (64.3)
University degree	5 (35.7)	5 (35.7)
Cancer type		
Prostate cancer	4 (28.6)	4 (28.6)
Breast cancer	3 (21.4)	3 (21.4)
Bladder cancer	2 (14.3)	2 (14.3)
Leukaemia	2 (14.3)	2 (14.3)
Lung cancer	1 (7.1)	1 (7.1)
Vulvar cancer	1 (7.1)	1 (7.1)
Cervical cancer	1 (7.1)	1 (7.1)
Lymphoma	1 (7.1)	1 (7.1)
Time since diagnosis in years (*SD; *range)	6.71 (5.55; 0–17)	6.71 (5.55; 0–17)
Prognosis		
Curative	2 (14.3)	2 (14.3)
Palliative	4 (28.6)	4 (28.6)
Currently cured	6 (42.9)	6 (42.9)
Unsure	2 (14.3)	2 (14.3)
Utilisation of psycho-oncological services		
Yes	11 (78.6)	11 (78.6)
No	3 (21.4)	3 (21.4)

^a^*SD S*tandard deviation

### Barriers and facilitators for the utilisation of POS

In the independent coding process of the data, a reliability score of *κ* = 0.68 (healthcare professionals) and *κ* = 0.79 (patients) was attained, indicating good interrater-agreement. Disagreements were discussed so that in the final coding round, conformity of all codes was achieved (*κ* = 1).

**Fig. 1:**
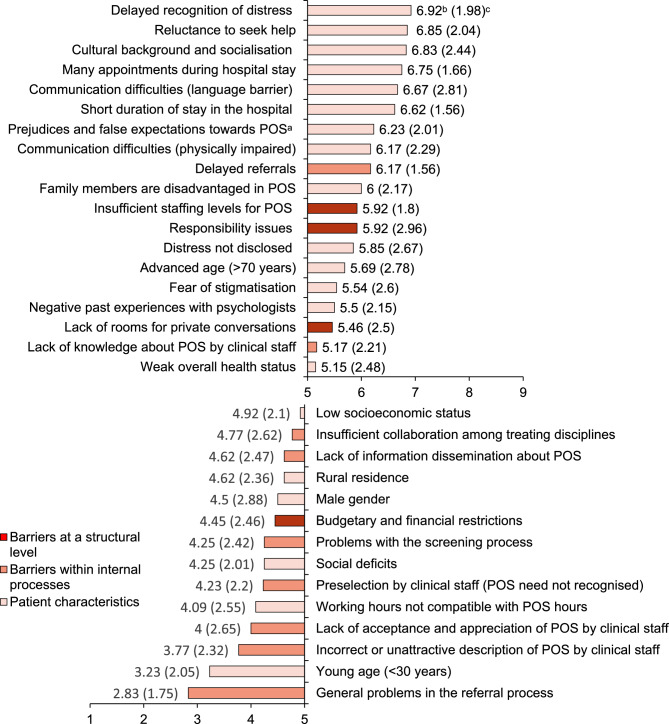
Barriers to psycho-oncological services as perceived by healthcare professionals (thematic collection Round1, *n*=27) and ranked according to their relevance (Round2, *n*=13). Note. The complete rating scale ranged from 1 to 9, with M>5 indicating agreement with the statement. ^a^POS=psycho-oncological services.^b^Mean. ^c^Standard deviation

**Fig. 2:**
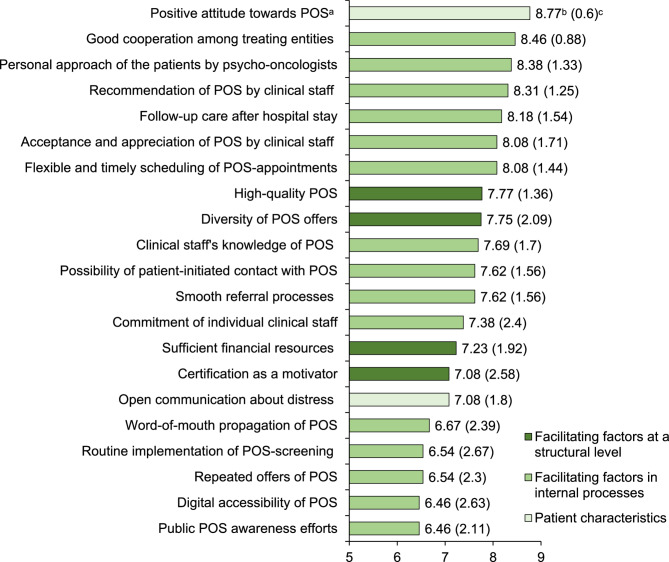
Facilitators to psycho-oncological services as perceived by healthcare professionals (thematic collection Round1, *n*=27) and ranked according to their relevance (Round2, *n*=13). Note. The complete rating scale ranged from 1 to 9, with M>5 indicating agreement with the statement.^a^POS=psycho-oncological services.^b^Mean. ^c^Standard deviation

**Fig. 3:**
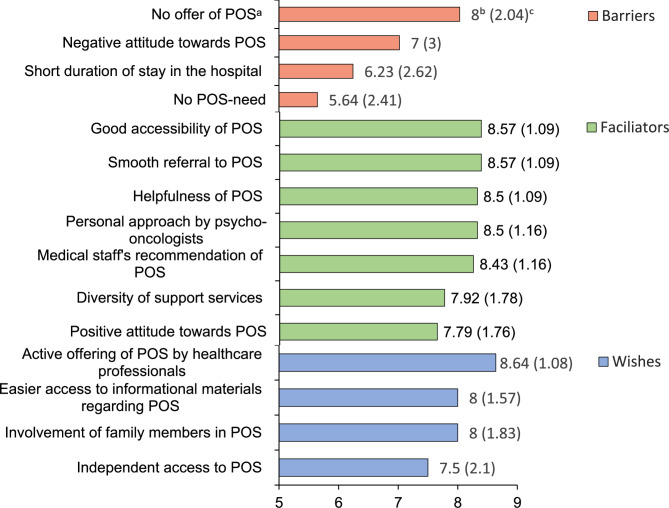
Barriers, facilitators and wishes for the utilisation of psycho-oncological services as perceived by cancer patients (thematic collection Round1, *n*=14) and ranked according to their relevance (Round2,*n*=14). Note. The complete rating scale ranged from 1 to 9, with M>5 indicating agreement with the statement.^a^POS=psycho-oncological services.^b^Mean. ^c^Standard deviation

69 different themes influencing the uptake of POS were identified in the qualitative analysis; 56 (81%) of them were rated as relevant (*M* > 5) in Round2. An overview of the results of the qualitative analysis (Round1) is shown in the Supplementary Material B. The results of the ratings of the different themes associated with the utilisation of POS (Round2) are presented in Figs. 1, 2 and 3.

#### Barriers

##### Healthcare professionals’ perspective

The hindering factors reported by healthcare professionals were grouped into three overarching themes according to their position in the healthcare system: structural factors at the hospital level, aspects related to internal processes, and factors at the patient level.

The highest ranked hindering factors in Round2 were patient-related, such as a delayed recognition of distress (“*The psychological consequences become apparent only upon returning to normal life*,* and the treatment and our services are already completed*.“, H_5_; “*Strains that arise later in the course*,* for example*,* due to side effects or complications after surgery*,* are sometimes overlooked*”, H_24_), reluctance to seek help (“*There is still a reluctance to seek support*.“, H_11_), cultural background and socialisation (e.g., not being familiar with the concept of psychosocial support offers or not having confidence in it) and negative attitudes towards POS such as prejudices and false expectations towards POS or fear of stigmatisation (“*Doubts about the effectiveness of psycho-oncology– Talking won’t make it any better now–*,* or reservations about psychological support or psychologists and fear of stigmatisation: societal*,* but also sometimes towards medical personnel– then nobody will take me seriously here anymore.*“, H_8_; *“Prejudices– e.g. I have cancer*,* but I’m not crazy.“*, H_5_), having many appointments during their hospital stay (*“Sometimes*,* the timing makes it difficult: procedures*,* examinations*,* treatments*, etc.,* often place a significant time and energy burden on patients*,* so it is understandable that rest and recovery are often desired afterwards*.“, H_9_) or, in general, a short stay in the hospital (“*In the inpatient setting*,* it is often difficult to reach patients with short hospital stays and numerous examinations*.“, H_24_).

On the level of internal processes, too late referrals (“*Sometimes patients were already discharged when the psycho-oncologists learned about their distress.“*, H_17_) and insufficient knowledge of clinical staff about psycho-oncology (“*At times*,* the (assistant) doctors seem to have no idea what we actually do and for which situations we can be called.“*, H_9_); *“Due to fluctuation in staff (nurses and doctors)*,* the POS process is not always equally familiar or integrated into the work*.“, H_20_) were seen as hindering the utilisation of POS.

At the structural level of the hospital, staff shortages (“*The staffing level of psycho-oncologists is not sufficient to make initial contact with every patient and*,* where necessary and needed*,* to establish more intensive contact*.“, H_12_), unclear responsibilities *(“At the time of diagnosis*,* the patients are in an unclear intermediate setting (outpatient/inpatient) and do not know about the PO offer and/or cannot access it (billing and responsibility problem!)”*, H_20_), and lack of rooms for private conversations (“*There is a lack of rooms on the wards where a protected discussion can take place. Often the conversations take place in the patient’s shared room*.“, H_16_; *“It is almost a miracle that despite these conditions*,* helpful conversations still happen from time to time*”, H_19_) were deemed particularly significant.

Of the 33 themes concerning barriers identified in Round1 60.6% were rated as relevant in the subsequent Round2.

##### Patients’ perspective

The most prominent barrier was the absence of a psycho-oncological support offer ("*I did not have this offer in the hospital; it was not available, but I needed it.*", P_2_), followed by negative attitudes towards psychological support (e.g. false expectations *“A chaplain with comforting words is not what I need for myself*.", P_11_), a short duration of the hospital stay, and no need for psychosocial support or relying on other sources of support (e.g. in the family system).

#### Facilitators

##### **Healthcare professionals’ perspective**

All facilitating factors that healthcare professionals reported in Round1 were rated as relevant in Round2. The highest-ranked facilitating factor was on the patient level: patients'positive attitudes towards POS. Concerning internal processes, the following aspects were considered essential: effective cooperation among healthcare disciplines ("*Years of established collaboration. Especially the nursing staff appreciates our work and makes efforts to point out patients in need and prepare consultation requests almost'ready-to-use,'thereby reducing the likelihood of these requests being'forgotten'on the physicians'side. With certain doctors, we have a very good cooperation*.", H_4_), personal introduction of psycho-oncologists to patients ("*it works wonders when they actually meet the person who offers the service*", H_8_;"*The personal contact with patients and their families can compensate for prejudices and negative experiences in surprisingly many cases*.", H_9_), recommendations or referrals for POS by clinical staff *("If the medical staff recommends the POS to a patient, it is like a prescription and is considered meaningful and important by the patient, resulting in a higher likelihood of utilisation", *H_1_), follow-up care and support offers after the hospital stay, acceptance and appreciation of POS within the hospital, as well as timely and flexible scheduling. Among the identified structural factors, high-quality and diverse psycho-oncological services (e.g., group-specific or low threshold offers) achieved the highest mean scores.

##### **Patients’ perspective**

Patients emphasised the relevance of good accessibility and referral to POS *("In my case, everything went well. In the beginning, at the acute hospital, the psycho-oncologist was assigned to me without me having to ask for it. Later, I was allowed to decide for myself and contact her as needed. I think that was optimal."*, P_14_), followed by a helpful offer, a personal approach by psycho-oncologists ("*It was a relief that someone approached me, and I didn't have to reach out to someone myself. This significantly reduced my hesitation to deal with it*.", P_1_), and recommendations from clinical staff *("The advice from the treating doctor was very important"*, P_6_). These findings were consistent with the patients'expressed wishes, which included easier access to informational materials *("But I had to look for the flyer on the ward myself. I would have preferred it if the doctor had a flyer with him directly.", *P_6_), involvement of family members, and an active approach of the POS staff as well as the option to self-refer to POS ("*If one could contact the POS oneself and not simply be overrun with a conversation offer*.", P_4_).

## Discussion

This study identified barriers and facilitators for the utilisation of POS in German hospitals, based on the perspectives of both healthcare professionals and cancer patients. Using a mixed-methods design with a two-round Delphi-inspired feedback process, we examined these factors in the context of existing clinical structures and pathways to POS.

The reported facilitating aspects by the participants aligned with the elements of the recommended clinical pathway to POS but also extended beyond them. While there was agreement on the importance of information provision, the significance of screening was rated lower than anticipated. Additionally, a universal access route to POS seems challenging since patients reported contrasting access preferences.

A prominent problem reported by patients was not receiving a POS-offer even if they wished for it. Possible reasons mentioned by the healthcare professionals could be found on a structural hospital level (financial restrictions and staff shortages) and the level of internal processes (e.g., delayed referrals to POS or lack of knowledge about the benefits of POS by the clinical staff). Facilitating factors, on the other hand, included recommendations of POS by the clinical staff, personal introductions of the psycho-oncological team, easy access to informational materials and POS being an established and well-integrated part of the hospital care system. For patients, flexible access to POS and good quality of the services encouraged uptake. Besides contextual factors, the expert panel identified certain patient groups as disadvantaged in the uptake of POS (e.g., patients with negative attitudes towards psychological services, reluctance to seek help, advanced age, language barriers or time constraints). From the point of view of healthcare professionals, these patient-related characteristics were rated as very relevant in determining the uptake of POS; patients tended to rate the structural aspects as more important.

While the decision to use POS ultimately lies with the patient, it is essential to minimise barriers and ensure accessibility to all who could benefit by creating equitable and easily accessible pathways to the service. The expert panel proposed measures to enhance POS-access, including offering educational trainings for clinical staff to encourage referrals and recommendations, providing follow-up care for patients with short hospital stays or delayed distress, and tailoring services for structurally disadvantaged groups.

Overall, the perspectives of healthcare professionals and cancer patients can be well integrated. While their viewpoints and focal areas differed to some extent, their observations converged in several key aspects. Both groups emphasised the negative impact of external constraints, such as time limitations during hospital stays and negative attitudes toward psychological services, as barriers to POS utilisation. A substantial overlap emerged in the identified facilitators: both patients and professionals highlighted the importance of a personal approach by psycho-oncologists, the perceived high quality and helpfulness of the services, and the role of smooth organisational referral processes in facilitating access. Additionally, an important insight derived from the patient perspective was the need for flexibility in accessing POS. Rather than a “one-size-fits-all” approach, patients expressed a preference for diverse access routes, suggesting that offering various ways to familiarise patients with the services could enhance service uptake.

The results of the present study complement the international literature. Often represented barriers are information deficits on the patients’ as well as on the staff’s side regarding POS [9, 17], and clinical staff not having enough time to screen and refer the patients appropriately [9, 17]. On the facilitating side, recommendation of POS by the clinical staff is considered very effective [9, 24–26], as well as a good integration of POS in routine care [27], which can accommodate patients’ wish for an easy and prompt access to the service [28]. Efforts to increase uptake of POS include implementing educational programs for clinical staff to enhance referrals and improve the screening process [29] and developing tailored informational materials and interventions for specific groups [30, 31].

### Strengths and limitations

The qualitative focus of this study should be taken into account when interpreting the results. While this approach cannot claim representativeness, its strength is to include a broad range of perspectives and allow contrasting opinions, thus representing individual preferences. The precise guideline-based content analysis and weighing of the different aspects in Round2 aimed to condense the extensive data into relevant findings using a mixed-methods approach.

However, several limitations must be considered. While the study successfully integrated diverse perspectives by including both healthcare professionals and patients across different hospital settings, greater diversity among professional groups would have been beneficial. Specifically, most participating healthcare professionals were psycho-oncologists, while nurses were not represented, leaving out an important group that might have identified additional influencing factors regarding the uptake of POS. Similarly, limitations may also apply to the patient sample: Our study design and recruitment via self-help groups likely resulted in a pre-selected sample, potentially underrepresenting disadvantaged groups such as individuals with lower educational levels or non-German speakers. Additionally, the snowball sampling approach may have reinforced these limitations by introducing selection bias and reducing diversity across professional and patient groups.

Another limitation is the attrition of participants from Round1 to Round2. While the primary focus of the study was on the qualitative insights gathered in the first round, the second round primarily aimed to prioritise the identified factors. However, as only 65.9% of the original participants completed the second survey, the prioritisation should be interpreted as an initial ranking of the facilitating and hindering factors within the clinical pathway to POS.

### Implications for future research and clinical practice

The results of this study could serve as a foundation for larger-scale quantitative surveys on facilitators and barriers to POS in diverse contexts (with Round2’s comprehensive collection of various barriers and facilitators serving as a reference). It could also be expanded to an international context, as our findings are limited to the German perspective. Additionally, future research could focus on marginalised groups using tailored designs to explore their specific needs within the clinical pathway to POS.

The findings from this study can be directly applied to clinical practice by offering healthcare institutions a framework for evaluating their current processes. By identifying the barriers and facilitators outlined in the study, institutions could pinpoint areas for improvement and enhance the aspects that support patient access to POS. Optimizing these aspects could ultimately contribute to higher utilisation of POS.

## Conclusion

Despite the existence of a clinical pathway to POS in Germany, a discrepancy remains between the apparent need for these services and their actual uptake by cancer patients. In this mixed-methods study, healthcare professionals in oncology and cancer patients identified 69 factors influencing POS utilisation, 81% of which were rated as relevant in the second survey round. Key factors included staff recommendations, direct introductions by psycho-oncologists, level of integration and acceptance of POS within hospital structures, effective information dissemination, and flexible access routes to accommodate varying patient needs. Additionally, certain patient groups were identified as more difficult to reach.

These findings can, on one hand, serve as a foundation for further research to examine common barriers and facilitators in larger, more representative contexts, and on the other hand, provide a basis for evaluating and optimising clinical pathways across different hospital settings to enhance accessibility and potentially increase POS utilisation.

## Supplementary Information

Supplementary Material 1.

Supplementary Material 2.

## Data Availability

The data supporting this study’s findings are available on request from the corresponding author.

## Abbreviation

POS Psycho: Oncological services
