# Supplementary material for: Barriers and facilitators for the utilisation of psycho-oncological services in German hospitals as perceived by patients and healthcare professionals: a mixed-methods study
Source: BMC Health Serv Res. 2025 Jul 1;25:851. doi: 10.1186/s12913-025-13053-5 (PMC12220343; doi:10.1186/s12913-025-13053-5)
Supplement: Supplementary file 1 — Supplementary Material 1. [file 12913_2025_13053_MOESM1_ESM.docx]

**Supplementary Material A: Provision of the Employed Surveys**

This supplement provides the four different surveys (two rounds, each with two different versions) which were the basis of the study “Barriers and Facilitators for the Utilisation of Psycho-Oncological Services in German Hospitals as Perceived by Patients and Healthcare Professionals: a Mixed-Methods Study”.

Note: The original surveys were conducted in German, the provided surveys are translated versions.

Overview

[Survey: Round 1 – Health Care Professionals‘ Version 1](#_Toc163196129)

[Survey: Round 1 – Patients‘ Version 3](#_Toc163196130)

[Survey: Round 2 – Health Care Professionals‘ Version 5](#_Toc163196131)

[Survey: Round 2 – Patients‘ Version 9](#_Toc163196132)

# Survey: Round 1 – Health Care Professionals‘ Version

**Structure of the Survey**

1. Information about the study and procedures
2. Questions regarding sociodemographic information as well working experience
3. Questions about the clinical pathway to psycho-oncological services
   *(provided in full length below)*
4. Core questions about usage of psycho-oncological services and factors influencing its uptake *(provided in full length below)*

**2) Questions Regarding the Clinical Pathway to Psycho-Oncological Services**

| Item | Response options |
| --- | --- |
| How are patients in your department informed about psycho-oncological services? | Flyer Website Discussion with doctors Discussion with nurses Psycho-oncological staff directly approaches patients Other: |
| Does your department conduct screening for psycho-oncological distress (e.g., in questionnaire form)? | Yes No I don't know |
| - *If yes to screening utilisation:*   Which screening instrument do you use? | Open response format |
| How often is the screening instrument used with a patient? | Once Occasionally In regular intervals At every appointment I don't know Not at all |
| Do you use any other methods besides a screening instrument to assess the psychosocial distress of your patients? | Yes No |
| - *If yes:*   What other approaches do you use to assess the psychosocial distress of your patients besides the screening instrument? | *Open response format* |
| - *If no to screening utilisation:*   Do you utilise alternative methods to assess the psychosocial distress of your patients? | Yes No |
| What other approaches do you use to assess the psychosocial distress of your patients? | *Open response format* |
| What are the next steps after identifying psychosocial distress? | *Open response format* |

**3) Core Questions: Factors Facilitating or Hindering the Utilisation of Psycho-Oncological Services**

| Item | Response options |
| --- | --- |
| Thank you for providing information about the current treatment pathway for psycho-oncological support in your facility.  Below are three open-ended questions where we kindly ask you to describe your experiences with potential problems and challenges in accessing psycho-oncological services, as well as what you believe facilitates or promotes service utilisation. These questions are the core focus of this study, so we appreciate your time in providing detailed responses. As these questions are based on your expertise and experiences, there are no right or wrong answers. After these three open-ended questions, the survey will be complete. | - |
| What aspects and circumstances in your department hinder the utilisation of psycho-oncological services or lead to not all individuals accessing the service who could benefit from it?  (Problem Description) | *Open response format* |
| Do you perceive any groups of people as disadvantaged in accessing psycho-oncological services (e.g., based on their gender, nationality, insurance status)? Which groups are these and why? How could this issue be addressed? | *Open response format* |
| What circumstances in your department contribute to the increased uptake of psycho-oncological services?  (What is going well?) | *Open response format* |

# Survey: Round 1 – Patients‘ Version

**Structure of the Survey**

1. Information about the study and procedures
2. Questions regarding sociodemographic information as well as cancer diagnosis and treatment
3. Questions about the clinical pathway to psycho-oncological services
   *(provided in full length below)*
4. Core questions about usage of psycho-oncological services and factors influencing its uptake *(provided in full length below)*

**2) Questions Regarding the Clinical Pathway to Psycho-Oncological Services**

| Item | Response options |
| --- | --- |
| With the following questions, we aim to understand the current clinical pathways to psycho-oncological services in various wards and clinics and what is perceived as good about it, and where improvements could be made.  There are no right or wrong answers to the following questions; we are interested in your personal opinions and experiences. | - |
| Were you informed about the possibility of psycho-oncological support?  (Psycho-oncological services = supportive services for coping with the psychological burden often associated with cancer) | Yes No Maybe / I don't know |
| How were you informed about the psycho-oncological services by your treatment team at the hospital? | Flyer  Website  Discussion with doctor  Discussion with nurse  Approached by a psycho-oncologist  Other: |
| Did your hospital treatment team test you for psychological distress in connection with your cancer (e.g. by means of a questionnaire)? | Yes No I don't know |
| - *If yes:*   How often have you filled out this questionnaire? | Once  Occasionally  At regular intervals  At every appointment |
| Has anyone else asked you about your emotional well-being or support needs in this area? | Yes  No |
| - *If yes:*   By whom or what were you asked? | *Open response format* |
| If you had the wish or need for psycho-oncological support, how did the referral to a counseling service work?  Please describe the referral process. | *Open response format* |

**3) Uptake of Psycho-Oncological Services and Factors Facilitating or Hindering the Uptake**

| Item | Response Option |
| --- | --- |
| Personal use of psycho-oncological services | I have received supportive counseling sessions with a psycho-oncologist.  I have not received supportive counseling sessions with a psycho-oncologist. |
| - *If no use of POS:*   I have not received supportive counseling sessions with a psycho-oncologist. | I would have appreciated supportive counseling sessions to discuss my cancer diagnosis and current life situation.  I did not feel the need for, or declined, supportive counseling sessions regarding my cancer diagnosis. |
| Here are the two final open-ended questions, focusing on what, in your view, facilitates or hinders the utilisation of psycho-oncological support services in the hospital. Once again, we are interested in your personal opinions and experiences, so there are no right or wrong answers. Since these two questions are the most crucial for our study, we would greatly appreciate it if you could provide detailed responses to both. | - |

- *The core questions for patients were tailored to their previous use of psycho-oncological services, resulting in three subgroups: users, involuntary non-users and deliberate non-users.*

Core questions for patients who indicated having used psycho-oncological services (users):

| Item | Response Option |
| --- | --- |
| What circumstances in the hospital led you to use the psycho-oncological services? (What went well?) | *Open response format* |
| What could have made it easier for you to access the psycho-oncological services in the hospital? (Possible issues and suggestions for improvement) | *Open response format* |

Core questions for patients who indicated not having used psycho-oncological services but would have appreciated it (involuntary non-users):

| Item | Response Option |
| --- | --- |
| Why were you unable to access the psycho-oncological support services? What was lacking in the hospital processes?  (Description of the problem) | *Open response format* |
| What should have been different in the hospital so that you could have taken advantage of the psycho-oncological services? What would you have wished for?  (Suggestions for improvement) | *Open response format* |

Core questions for patients who indicated not having used psycho-oncological services and had no need or wish for it (deliberate non-users):

| Item | Response Option |
| --- | --- |
| Why did you not feel the need for psycho-oncological support? | *Open response format* |
| Why did you not wish to receive psycho-oncological support? | *Open response format* |

# Survey: Round 2 – Health Care Professionals‘ Version

**Structure of the Survey**

1. Information about the status of the study und procedures *(excerpt is provided below)*
2. Questions regarding sociodemographic information as well working experience
3. Ranking of the hindering and facilitating aspects for the uptake of psycho-oncological services on the different levels *(provided in full length below)*

**0) Information about the Study and Procedure**

Dear participants of the survey "Utilisation of Psycho-Oncological Services in Hospitals",

First and foremost, we would like to extend our sincere thanks for your comprehensive reports on your perspectives regarding the utilisation of psycho-oncological services! Through a qualitative analysis method, we have uncovered many intriguing aspects and commonalities in your responses.
Overall, we have analysed the open-ended responses from 27 healthcare providers (primarily from the psycho-oncology field as well as doctors) and 14 cancer patients. In the following, we will present the points mentioned by the healthcare providers regarding the hindering and facilitating factors of utilisation. You will then have the opportunity to assess the extent to which each aspect applies to your work environment and how relevant you consider it to be. The aim of this second survey round is to obtain a weighting of all mentioned barriers and facilitators.

**2) Ranking of the Aspects Influencing the Uptake of Psycho-Oncological Services on Different Levels**

Hindering Factors for Utilisation of Psycho-Oncological Services

First, aspects that hinder the utilisation of psycho-oncological services will be addressed. We have divided your responses in this area into structural issues at the hospital level, problems in internal processes, and patient characteristics.

*Barriers at a Structural Hospital Level*

| Item - start | Item - ending | Response format |
| --- | --- | --- |
| For the uptake of psycho-oncological services (in the hospital), I perceive as hindering... | … inadequate facilities (e.g., lack of quiet or private spaces in wards, conversations must take place in multi-bed rooms). | *Scale from 1 to 9 with the following verbal anchors 1 (strongly disagree), 5 (partially agree), 9 (strongly agree).* |
|  | … financial constraints (e.g., billing difficulties, limited budget for psycho-oncological services). |  |
|  | … problems with responsibility allocation within the hospital (e.g., patients on certain wards or in intermediate stages [outpatient/inpatient] are underserved). |  |

*Barriers in Internal Processes*

| Item - start | Item - ending | Response format |
| --- | --- | --- |
| For the uptake of psycho-oncological services (in the hospital), I perceive as hindering... | … lack of information dissemination about psycho-oncological services (e.g., services are unknown, patients are not made aware of it). | *Scale from 1 to 9 with the following verbal anchors 1 (strongly disagree), 5 (partially agree), 9 (strongly agree).* |
|  | … insufficient collaboration (e.g., between psycho-oncology and other clinical staff, lack of exchange). |  |
|  | … lack of acceptance and appreciation of psycho-oncological services by other clinical staff (e.g., lack of focus or interest among other clinical staff in addressing non-physical ailments). |  |
|  | … lack of knowledge about psycho-oncological services among other clinical staff (e.g., regarding the content of the services or situations for which they can be used). |  |
|  | … preselection of patients by other clinical staff or failure to recognise the need (e.g., misjudgment of the need, lack of objectivity and fairness). |  |
|  | … problems with the screening (e.g., screening instrument not distributed/conducted, results not communicated). |  |
|  | … general problems in referral or registration to a psycho-oncological consultation (e.g., the consultation registrations are not processed correctly). |  |
|  | … late referral (e.g., referral just before discharge). |  |

*Barriers: Patient-Related Characteristics*

| Item - start | Item - ending | Response format |
| --- | --- | --- |
| Groups of people where I perceive lower utilisation of psycho-oncological services are individuals who... | …do not openly show or communicate their distress (e.g., are calm and reserved). | *Scale from 1 to 9 with the following verbal anchors 1 (strongly disagree), 5 (partially agree), 9 (strongly agree).* |
|  | …whose distress becomes apparent only after hospitalisation. |  |
|  | …have social deficits or personality disorders. |  |
|  | …are not proficient in German (e.g., have a different mother tongue). |  |
|  | …cannot speak properly due to physical impairment. |  |
|  | …have a poor general condition. |  |
|  | …have a different socialization regarding psychological support (e.g., due to a different cultural background). |  |
|  | …are male. |  |
|  | …have a low socioeconomic status. |  |
|  | …live in rural areas. |  |
|  | …have a short hospital stay. |  |
|  | …have a high appointment frequency in the hospital. |  |
|  | …cannot utilise psycho-oncological services due to their occupation (time constraints). |  |
|  | …have negative past experiences with psychologists. |  |
|  | …have prejudices and false expectations regarding psychological services. |  |
|  | …are hesitant to seek help. |  |
|  | …fear stigmatisation through seeking psychological help. |  |
|  | …are elderly (over 70 years). |  |
|  | …are young (under 30 years). |  |
|  | Family members of cancer patients find it more difficult to utilise psycho-oncological services in general. |  |

Facilitating Factors for Utilisation of Psycho-Oncological Services

The aspects the panel described as facilitating for the uptake psycho-oncological services are listed below. We have categorised the topics into three major areas: Facilitating factors at a structural hospital level, in internal processes, and patient characteristics.

*Facilitating Factors at a Structural Hospital Level*

| Item - start | Item - ending | Response format |
| --- | --- | --- |
| For the uptake of psycho-oncological services (in the hospital), I perceive as facilitating... | …the hospital’s efforts to become certified as a cancer center (e.g., perception of the certification as a motivator). | *Scale from 1 to 9 with the following verbal anchors 1 (strongly disagree), 5 (partially agree), 9 (strongly agree).* |
|  | …good financial resources of the clinic regarding psycho-oncological services. |  |
|  | …high quality of psycho-oncological services (e.g., high qualification of psycho-oncologists, perceived conversations as helpful by patients). |  |
|  | …diverse offerings (e.g., also low-threshold offerings, specialised offerings). |  |

*Facilitating Factors in Internal Processes*

| Item - start | Item - ending | Response format |
| --- | --- | --- |
| For the uptake of psycho-oncological services (in the hospital), I perceive as facilitating... | …recommendations of psycho-oncological services by other clinical staff. | *Scale from 1 to 9 with the following verbal anchors 1 (strongly disagree), 5 (partially agree), 9 (strongly agree).* |
|  | …good public relations work (e.g., good availability of informational materials with contact information). |  |
|  | …word-of-mouth promotion (by satisfied patients). |  |
|  | …routine use of the screening instrument. |  |
|  | …established collaboration and mutual exchange (e.g., between the psycho-oncology team and other clinical staff, between departments, team-internal). |  |
|  | …acceptance and appreciation of psycho-oncological services within the treatment team (including medical and nursing staff). |  |
|  | …knowledge and awareness of psycho-oncological services among clinic staff. |  |
|  | …individual efforts. (e.g., utilisation is driven forward by the efforts of individuals) |  |
|  | …well-functioning referral processes. |  |
|  | …repeated conversation offers. |  |
|  | …aftercare and offerings of psycho-oncological services after hospitalisation. |  |
|  | …possibility of contact to psycho-oncological services from the patient's side. |  |
|  | …(digital) accessibility to psycho-oncological services (e.g., possibility for video and telephone conversations). |  |
|  | …flexible and timely scheduling of appointments. |  |

*Facilitating Patient-Related Characteristics*

| Item - start | Item - ending | Response format |
| --- | --- | --- |
| Groups of people where I perceive a higher utilisation of psycho-oncological services are individuals who... | …have a positive attitude towards psychological services. | *Scale from 1 to 9 with the following verbal anchors 1 (strongly disagree), 5 (partially agree), 9 (strongly agree).* |
|  | …whose distress is visible outwardly (e.g., through communication of distress). |  |

# Survey: Round 2 – Patients‘ Version

**Structure of the Survey**

1. Information about the status of the study und procedures *(excerpt provided below)*
2. Questions regarding sociodemographic information as well as cancer diagnosis and treatment
3. Ranking of the aspects influencing the uptake of psycho-oncological services *(provided in full length below)*

**0) Information about the Status of the Study und Procedures**

The aspects collected in the first round are now presented, sorted by barriers, facilitating factors, and wishes or suggestions for improvement. Your task is to assess the relevance of each aspect. You can indicate your agreement on a scale from 1 (strongly disagree) to 9 (strongly agree).
We kindly ask for a general assessment from your perspective. This way, regardless of your personal prior experiences, you can provide an evaluation for all aspects.

**2) Ranking of the Aspects Influencing the Uptake of Psycho-Oncological Services**

Barriers

Please assess the following barriers for how hindering they are (regarding the uptake of psycho-oncological services in the hospital), meaning they make the uptake more difficult.

| Item - start | Item - ending | Response format |
| --- | --- | --- |
| The uptake of psycho-oncological services in the hospital is hindered... | …when no offer is made (e.g., patients are not made aware of the psycho-oncological services). | *Scale from 1 to 9 with the following verbal anchors 1 (strongly disagree), 5 (partially agree), 9 (strongly agree).* |
|  | … when the duration of hospital stay is too short. |  |
|  | …when patients have a negative attitude towards psychological offers (e.g., they do not consider the offer helpful or meaningful). |  |
|  | …when patients do not need the services because they receive other support (e.g., from family). |  |

Facilitating Factors

Please assess the following facilitating factors for how helpful they are (regarding the uptake of psycho-oncological services in the hospital), meaning they make the uptake easier.

| Item - start | Item - ending | Response format |
| --- | --- | --- |
| The uptake of psycho-oncological services in the hospital is facilitated... | … when patients are personally addressed by a psycho-oncologist. | *Scale from 1 to 9 with the following verbal anchors 1 (strongly disagree), 5 (partially agree), 9 (strongly agree).* |
|  | … when patients receive a referral to the service from other clinical staff (doctors, nurses, etc.). |  |
|  | … when internal and/or external referrals work well.  (e.g., smooth process from offer to conversation, assistance in finding external services) |  |
|  | … when the services or the psycho-oncologists are easily accessible (e.g., uncomplicated contact options, availability beyond the hospital stay). |  |
|  | … when the psycho-oncological services are diverse.  (e.g., male and female psycho-oncologists, varied course offerings, low-threshold services such as oncology cafes) |  |
|  | … when psycho-oncological conversations are helpful (e.g., experienced as helpful by patients). |  |
|  | … when patients have a positive attitude towards psychological offers (e.g., through positive testimonials from other patients). |  |

Wishes and Suggestions for Improvement

Please assess the following improvement suggestions for how desirable they are (regarding the uptake of psycho-oncological services in the hospital).

| Item - start | Item - ending | Response format |
| --- | --- | --- |
| Regarding psycho-oncological services in the hospital, it would be desirable... | … if patients were actively approached by clinical staff (doctors, nurses, etc.). | *Scale from 1 to 9 with the following verbal anchors 1 (strongly disagree), 5 (partially agree), 9 (strongly agree).* |
|  | … if patients received information about the service from clinical staff (doctors, nurses, etc.). |  |
|  | … if patients could approach the service or psycho-oncologists autonomously. |  |
|  | … if information material was easily accessible (e.g., presence of a flyer, distribution of informational material). |  |
|  | … if family members were involved in psycho-oncological services. |  |
