# Supplementary material for: Barriers and facilitators for the utilisation of psycho-oncological services in German hospitals as perceived by patients and healthcare professionals: a mixed-methods study
Source: BMC Health Serv Res. 2025 Jul 1;25:851. doi: 10.1186/s12913-025-13053-5 (PMC12220343; doi:10.1186/s12913-025-13053-5)
Supplement: Supplementary file 2 — Supplementary Material 2. [file 12913_2025_13053_MOESM2_ESM.docx]

**Supplementary Material B:**

**Results of the Qualitative Analysis of Round1**

| **Supplementary Table B1** *Results of the Qualitative Analysis: Structural Factors Influencing the Uptake of Psycho-oncological Services in a Hospital Setting as Perceived by Healthcare Professionals* | |
| --- | --- |
| **Overarching themes** | **Themes** (with subthemes, if existing) |
| **Hindering factors at a structural level (18)^a^** | Insufficient staffing levels for POS^b^ (9)^a^  Lack of rooms for private conversations (4)  Budgetary and financial restrictions (3)  Responsibility issues (2) |
| **Hindering factors within internal processes (51)** | Lack of POS-need assessment (20)   - Problems with the screening process - Preselection by clinical staff (POS need not recognised)   Lack of information dissemination about POS (8)  Insufficient knowledge of POS by medical staff (8)   - Incorrect or unattractive description of POS by clinical staff   Issues with referral to POS (7)   - Delayed referrals   Lack of acceptance and appreciation of POS by medical staff (5) Insufficient collaboration among treating disciplines (3) |
| **Hindering factors related to patient characteristics (117)** | Communication difficulties (25)   - Communication difficulties due to language barrier - Communication difficulties due to physical condition   Negative attitudes towards POS (23)   - Prejudices and false expectations towards POS - Fear of stigmatisation - Negative past experiences with psychologists - Reluctance to seek help   Cultural background and socialization (12)  Time constraints for patients (11)   - Many appointments during hospital stay - Short duration of stay in the hospital - Working hours not compatible with POS hours   Male gender (10)  Weak overall health status (7)  Age (7)   - Young age (< 30 years) - Advanced age (> 70 years)   Distress not disclosed (7)  Low socioeconomic status (4)  Rural residence (4)  Delayed recognition of distress (2)  Social deficits (1)  Family members are generally disadvantaged in accessing POS (4) |
| **Facilitating factors at a structural level (13)** | Presence of good POS (11)   - High-quality POS - Diversity of psycho-oncological support services   Sufficient financial resources (1)  Certification as a motivator (1) |
| **Facilitating factors in internal processes (81)** | Good cooperation among treating entities (17)  Recommendation of POS by medical staff (12)  Good and flexible scheduling and accessibility of POS (12)   - Flexible and timely scheduling of POS-appointments - Repeated offers of POS - Digital accessibility of POS - Possibility of patient-initiated contact with POS - Follow-up care and support offers after hospital stay   Acceptance and appreciation of POS by clinical staff (10)  Personal approach of the patients by psycho-oncologists (7)  Clinical staff's knowledge about POS (5)  Involvement of individual treating professionals (5)  Public awareness efforts (4)  Routine implementation of a screening instrument for POS-need (4)  Word-of-mouth referrals (3)  Smooth referral processes (2) |
| **Facilitating factors related to patient characteristics (2)** | Positive attitude towards POS (1)  Open communication about distress (1) |
| *Note*: ^a^ Number of times the theme occurred in the data. ^b^ POS = psycho-oncological services. | |

| **Supplementary Table B2** *Results of the Qualitative Analysis: Structural Factors Influencing the Uptake of Psycho-oncological Services in a Hospital Setting as Perceived by Cancer Patients* | |
| --- | --- |
| **Overarching themes** | **Themes** (with subthemes, if existing) |
| **Barriers (6)^a^** | Patient characteristics (3)^a^   - No need for POS^b^ - Negative attitudes towards POS   No offer of POS (2)  Short duration of stay in the hospital (1) |
| **Facilitators (17)** | Smooth referral to POS (5)  Good accessibility of POS (3)  Helpfulness of POS (3)  Clinical staff's mention of POS (2)  Personal approach by psycho-oncologists (2)  Diversity of support services (1)  Patient characteristics (1)   - Negative attitudes towards POS |
| **Wishes (6)** | Active offering of POS by clinical staff (2)  Easier access to informational materials regarding POS (2)  Independent access to POS (1)  Involvement of family members in POS (1) |
| *Note*: ^a^ Number of times the theme occurred in the data. ^b^ POS = psycho-oncological services. | |
